# Assessment of human resources for health using cross-national comparison of facility surveys in six countries

**DOI:** 10.1186/1478-4491-7-22

**Published:** 2009-03-12

**Authors:** Neeru Gupta, Mario R Dal Poz

**Affiliations:** 1 Department of Human Resources for Health, World Health Organization, Geneva, Switzerland

## Abstract

**Background:**

Health facility assessments are being increasingly used to measure and monitor indicators of health workforce performance, but the global evidence base remains weak. Partly this is due to the wide variability in assessment methods and tools, hampering comparability across and within countries and over time. The World Health Organization coordinated a series of facility-based surveys using a common approach in six countries: Chad, Côte d'Ivoire, Jamaica, Mozambique, Sri Lanka and Zimbabwe. The objectives were twofold: to inform the development and monitoring of human resources for health (HRH) policy within the countries; and to test and validate the use of standardized facility-based human resources assessment tools across different contexts.

**Methods:**

The survey methodology drew on harmonized questionnaires and guidelines for data collection and processing. In accordance with the survey's dual objectives, this paper presents both descriptive statistics on a number of policy-relevant indicators for monitoring and evaluation of HRH as well as a qualitative assessment of the usefulness of the data collection tool for comparative analyses.

**Results:**

The findings revealed a large diversity in both the organization of health services delivery and, in particular, the distribution and activities of facility-based health workers across the sampled countries. At the same time, some commonalities were observed, including the importance of nursing and midwifery personnel in the skill mix and the greater tendency of physicians to engage in dual practice. While the use of standardized questionnaires offered the advantage of enhancing cross-national comparability of the results, some limitations were noted, especially in relation to the categories used for occupations and qualifications that did not necessarily conform to the country situation.

**Conclusion:**

With increasing experience in health facility assessments for HRH monitoring comes greater need to establish and promote best practices regarding methods and tools for their implementation, as well as dissemination and use of the results for evidence-informed decision-making. The overall findings of multi-country facility-based survey should help countries and partners develop greater capacity to identify and measure indicators of HRH performance via this approach, and eventually contribute to better understanding of health workforce dynamics at the national and international levels.

## Background

Human resources are a strategic capital in any organization, but particularly so in health and other service organizations that are highly dependent on their workforce. The functioning and growth of health systems depends on the availability of human resources and on the time, effort and skill mix provided by the workforce in the execution of its tasks [1,2]. There is growing international recognition that one of the key ingredients in achieving improved population health outcomes is an adequate and available health workforce [3,4]. At the same time, there is general consensus that human resources for health (HRH) have been a neglected component of health systems development in low-income and middle-income countries [5].

Many countries lack the human resources needed to deliver essential health interventions for a number of reasons, including limited production capacity, migration of health workers within and across countries, poor mix of skills and demographic imbalances. The formulation of national policies and plans in pursuit of health workforce development objectives requires sound information and evidence. Against the backdrop of increasing demand for information, building knowledge and understanding of the health workforce requires coordination across sectors. It is being increasingly recognized that cross-national comparisons provide opportunities for gaining insights into many HRH issues of major concern to many countries and learning how other countries have dealt successfully or otherwise with these issues [6].

Although a number of sources exist even in low-income countries that can potentially provide data relevant to health workforce analysis – including population- and facility-based censuses and surveys, as well as administrative and management records – information on health system personnel is often fragmented or incomplete. Health facility assessments are being increasingly used to measure and monitor indicators of health worker performance, but the global evidence base remains weak [7]. The diversity of methods and tools used to implement data collection means that considerable variability occurs in data coverage and quality, hampering comparability across and within countries and over time. For example, a previous analysis of health worker distribution using facility data from three developing countries acknowledged that the lack of a standardized occupational coding system to identify provider type resulted in difficulties in conducting cross-national comparisons [8].

To strengthen the evidence base on HRH from an international perspective, the World Health Organization (WHO) coordinated a series of facility-based assessments in six low-income and middle-income countries. The objectives were twofold: to inform the development of HRH policy within the countries and to test and validate the use of standardized survey instruments across different contexts. Four of the countries were located in Africa (Chad, Côte d'Ivoire, Mozambique and Zimbabwe), one in Asia (Sri Lanka), and one in the region of Latin America and the Caribbean (Jamaica). As seen in Table 1, the countries present a large diversity in basic demographic and health indicators, notably in terms of population size (from under 3 million in Jamaica to nearly 20 million in Mozambique), life expectancy (from 37 years in Zimbabwe to over 70 in Jamaica), and infant mortality (from under 20 deaths per thousand in Jamaica and Sri Lanka to 124 in Chad). The four African countries have been identified as having a critical shortage of skilled medical, nursing and midwifery personnel [9].

**Table 1:** Selected demographic and health indicators by country (around 2004)

	Income category*	**Population **(millions)	**Life expectancy at birth **(years)	Infant mortality rate** (‰)
**Chad**	Low	9.7	44.0	124
**Côte d'Ivoire**	Low	18.2	46.2	118
**Jamaica**	Lower-middle	2.7	70.9	17
**Mozambique**	Low	19.8	41.8	100
**Sri Lanka**	Lower-middle	19.6	74.7	12
**Zimbabwe**	Low	13.0	37.3	81

Sources: UNESCO Institute of Statistics Data Centre; World Bank World Development Indicators database, April 2007.

* Income category as classified by the World Bank according to 2006 Gross National Income (GNI) per capita.

** Infant mortality rate = Number of newborns dying under a year of age per thousand live births.

This paper presents the main findings of the six survey-based HRH assessments. In accordance with the assessment's overall objectives, the analysis here follows a two-pronged approach: in terms of the usefulness of the data collection tool for cross-country comparisons, and in terms of country-specific findings relevant for HRH policy and planning.

## Methods

The Assessment of Human Resources for Health was conducted in six countries between 2002 and 2004, with technical and financial support from WHO. A common approach was proposed to collect data by means of personal interview with a sample of facility-based health care providers on a number of topics, including professional qualifications, demographic characteristics, work activities, workplace conditions and remuneration [10]. An additional questionnaire at the level of the facility was designed to collect supplementary information on staffing distribution by location and other characteristics of place of work, and a third questionnaire was used to compile national-level information from health ministries and professional councils on regulation of health occupations.

The methodology drew on standardized questionnaires and guidelines for data collection and processing. In order for the eventual results to be comparable across countries, it was recommended that the sampling frames be compiled the same way in every setting, and that the questionnaires be filled the same way with each respondent. As such, standard training guidelines were provided by WHO for all field enumerators. Standard data entry software templates were also developed for all data entry operators, by means of the SPSS Data Entry Builder software program [11]. The instruments were translated to meet the language needs of some countries, but otherwise essentially unchanged. In particular, a pre-coded list of (presumed) occupational titles for facility staff was provided drawn from the International Standard Classification of Occupations (ISCO), a framework for enhancing comparability of labour statistics by means of grouping of jobs according to shared characteristics [12].

Data collection and processing were implemented in each country by a national collaborating agency: Centre de Support en Santé Internationale/Institut Tropical Suisse au Tchad (Chad), Ministère de la Santé (Côte d'Ivoire), Ministry of Health (Jamaica), Ministério da Saúde/Centro Regional de Desenvolvimento Regional Sanitário (Mozambique), Ministry of Health (Sri Lanka), and the University of Zimbabwe (Zimbabwe).

This analysis focuses on key results from the health care providers questionnaire (see Additional File 1). We present descriptive statistics on a number of policy-relevant indicators for monitoring and evaluation of HRH, including skill mix, age and sex distribution, educational attainment, institutional sector and labour market activity [13]. Where appropriate, additional quantitative and qualitative information compiled via the questionnaires on health facilities and regulation of health occupations as well as field reports from the national implementing agencies are used for country-specific contextual analysis.

The sample size of providers surveyed in each country is presented in Table 2. The final number of respondents ranged from 364 in Jamaica to 2354 in Sri Lanka. Based on the original guidelines, it was expected that the sample would be drawn using a stratified systematic random selection technique to include representation across each country's main regions, the different types of facilities (hospitals/health centres, public/private) and the various workforce domains (occupation, age group, sex, etc.).

**Table 2:** Sample size of health facilities and providers, Assessment of Human Resources for Health, 2002–2004

	**Number of sampled facilities**	**Number of surveyed health care providers**	**Mean number of providers surveyed per facility**
**Chad**	213	545	2.6
**Côte d'Ivoire**	313	709	2.3
**Jamaica**	214	364	1.7
**Mozambique**	195	737	3.8
**Sri Lanka**	222	3130	14.1
**Zimbabwe**	105	1026	9.8

(It may be noted that general information from a range of countries using different tools for assessment of facility-based service delivery points, including HRH, as well as news on international technical cooperation efforts and developments in strengthening facility-based data collection and use, is available on the web site of the International Health Facility Assessment Network [14].)

## Results

### Implementation of data collection

Despite efforts to reduce survey variances across countries by means of standardized data collection tools and approaches, considerable variations did occur in implementation due to specificities of national health systems as well as logistic, technical and sociopolitical reasons.

**Figure 1:**
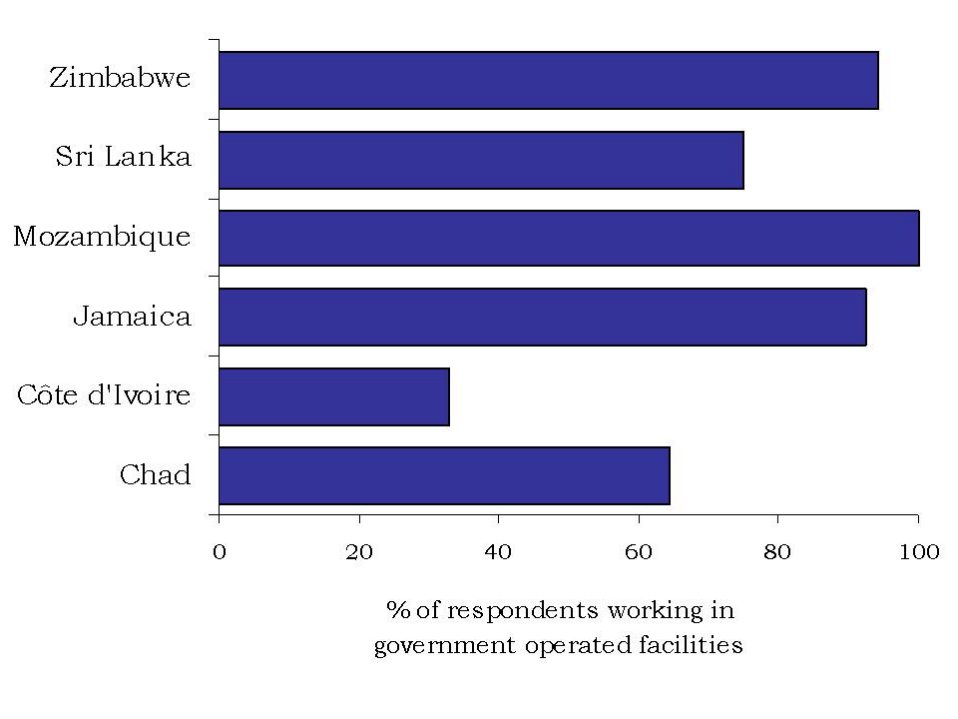
**Percentage of surveyed health care providers working in government-operated facilities**.

First, it must be recognized that in most countries the final survey samples was biased towards the public sector. In many countries the response rate of facilities and providers in the private sector was low. In Mozambique, for instance, although 45 private clinics had initially been identified and included in the sampling frame, the response rate was very low, and this despite repeated contacts from the field investigators, to the extent that the eventual results presented here have been limited to public sector providers alone (Figure 1). Among the main reasons cited for non-response to the survey were misconceptions about the purpose of the assessment (i.e. government inspection rather than research and policy purposes alone) and work overload. In Jamaica, only 5% of private physicians were surveyed. On the other hand, in Sri Lanka, a representative of the Independent Medical Practitioners Association was involved in the survey group from the initial planning stages. Data collection was more successful in the private sector in this context. The highest proportion of private providers interviewed was found in Côte d'Ivoire, where civil conflict and worsening socioeconomic conditions between 2002 and 2004 have been linked to exacerbated worker shortages and high levels of attrition in the public health sector [15].

A shortage of health personnel, and particularly certain highly skilled cadres, was a hindrance in some countries to meeting the original sampling design. In Mozambique, the sample of facilities was changed during the course of fieldwork in some areas because of an unanticipated lack of personnel available for interview. In Jamaica, a number of the smallest (Type 1) government-operated primary health centres were found to be closed on the day of the visit, so some types of other, larger health centres (Types 2–5) were oversampled instead. In Sri Lanka, the minimum number of providers to be interviewed per facility was increased to capture more workers in smaller facilities.

Limited information and communications technologies in some countries affected the survey implementation processes. In Jamaica, it was not possible to compile a master list of all currently employed health workers in government and private health facilities because much depended on the level of computerization of local management information systems. (The required information was eventually obtained during the course of fieldwork from the individual facilities or providers themselves.) A lack of email service at the Ministry of Health's Department of Human Resources in Côte d'Ivoire resulted in some delays in coordinating efforts and sharing knowledge. This situation was reflective of a widespread lack of information technology and telecommunications across the African region: a 2004 study conducted by the WHO Regional Office for Africa showed that 22% of health workforce departments in ministries of health in the region did not have computer facilities, 45% had no email access and 68% did not have a fax machine [16].

As previously mentioned, Côte d'Ivoire experienced civil conflict around the time of the survey. Fieldwork was delayed by several months from the original plans due to the sociopolitical crisis, and when eventually implemented the sampling was subject to significant modifications compared with the initial design. Some parts of the country were not covered, and the final sample of 313 facilities represented only 73% of the initial target – with relatively more private than public facilities captured, compared with the original plans (75% versus 68%). In Sri Lanka, some parts in the north and east of the country were excluded from the sampling frame due to long-standing civil conflict in those areas.

### Profile of the health workforce

Globally, the health workforce is characterized by a diversity of occupations and skills. However the specific mix varies greatly across contexts. While there is no international "gold standard" for an appropriate skill mix to meet the health needs of a given population, measuring this mix offers a means to assess the combination of categories of personnel at a specific time and identify possible imbalances related to a disparity in the numbers of various health occupations.

In all the six countries, nursing and midwifery personnel represented the largest group of facility-based workers surveyed (Table 3). In Jamaica, community health aides were also captured in this group, considered to be equivalent to auxiliary nurses. The share of physicians ranged from a high of 26% in Sri Lanka to some 5% to 7% in Chad, Mozambique and Zimbabwe. Few pharmacists or physiotherapists were found in any of the survey samples. The catch-all "other" category captured a very large share of workers in some countries, including a wide range of middle- and lower-level service providers (such as medical assistants, dental assistants, pharmaceutical auxiliaries and X-ray technologists) as well as health management and support staff (such as administrators and maintenance crews) needed to keep facilities running.

**Table 3:** Percentage distribution of the facility-based health workforce by occupation, Assessment of Human Resources for Health

	**Chad**	**Côte d'Ivoire**	**Jamaica**	**Mozambique**	**Sri Lanka**	**Zimbabwe**
Physicians	6	13	10	5	26	7
Nurses	14	28	27	37	36	46
Midwives	6	7	11	4	11	19
Auxiliary nurses	41	22	33	3	1	--
Auxiliary midwives	3	2	5	5	1	--
Pharmacists	1	4	2	1	4	1
Physiotherapists	<1	<1	1	<1	2	1
Other health workers	29	23	11	45	19	26

-- = no observations in survey sample

Note: Percentages may not sum to 100% due to rounding.

**Figure 2:**
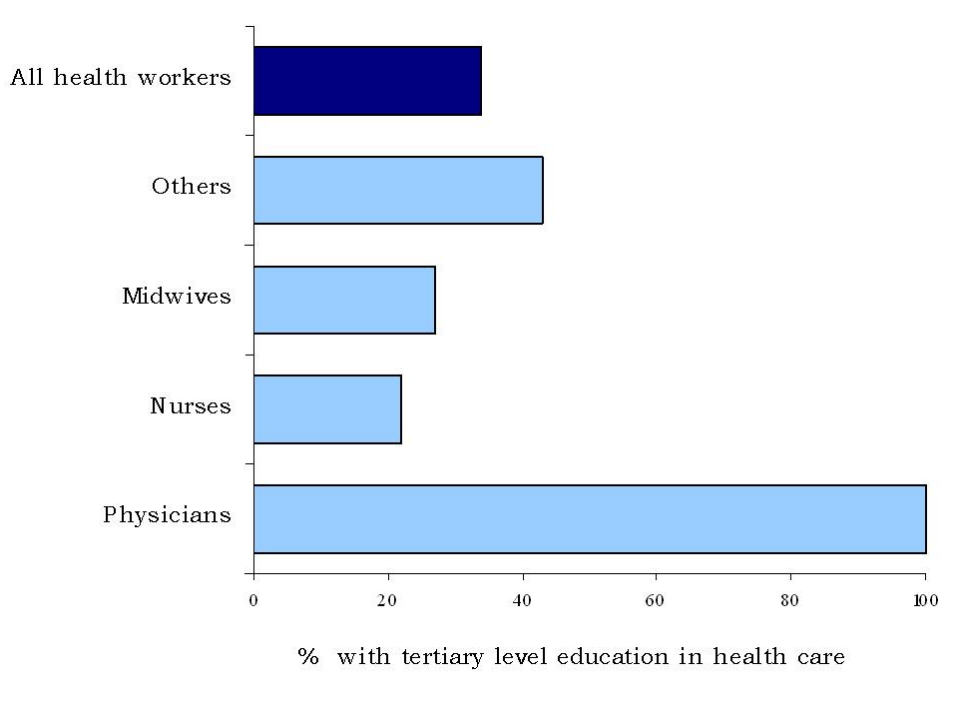
**Percentage of facility-based health workers with tertiary-level education, by occupation, Zimbabwe**.

As expected, along with wide differences in the workforce skill mix, variations were also observed in the level of professional education and training among surveyed health workers, both within and across countries. In Zimbabwe, while all physicians reported having a university education, only a quarter of nursing and midwifery personnel did so (Figure 2). In Jamaica, all the respondents reported having university or professional qualifications that enabled them to practise legally in the country, while in Chad only 15% of respondents had a university diploma in health care (results not shown). In Sri Lanka, national regulations require all health service providers except auxiliary nurses and midwives to receive their training at government-operated education institutions. In Mozambique, 42% of physicians reported having conducted their studies in another country.

**Figure 3:**
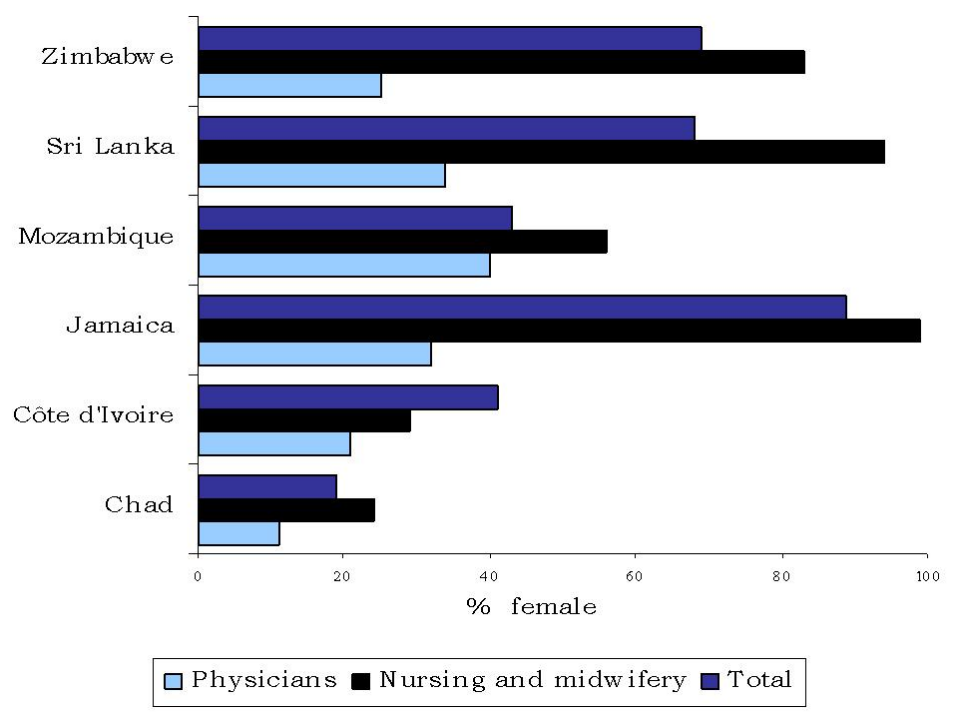
**Sex distribution of the facility-based health workforce, by occupation**.

In Jamaica, Sri Lanka and Zimbabwe, women comprised at least 70% of the health workforce (Figure 3), making them indispensable as contributors to the delivery of health care services in these countries. In contrast, in Chad only 19% of surveyed health workers were women. In all the countries, women tended to be concentrated among nursing and midwifery personnel and mostly lower-level occupations, and were poorly represented among physicians. In Côte d'Ivoire and Mozambique a majority of nursing personnel were male, but midwifery personnel were predominantly women (results not shown).

**Figure 4:**
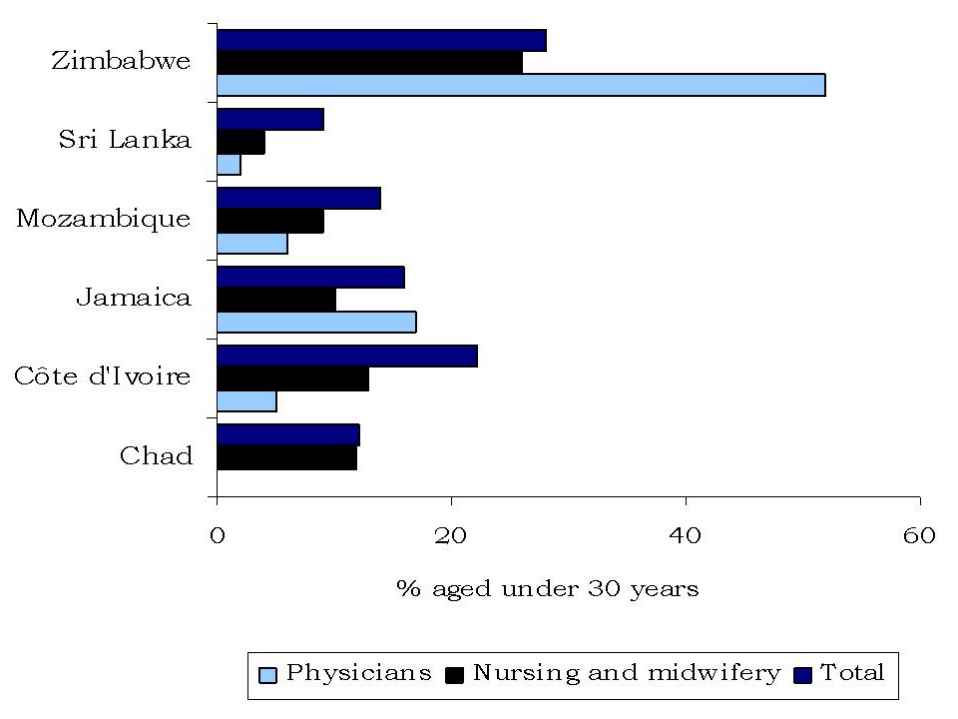
**Age distribution of the facility-based health workforce, by occupation**.

The age distribution of the health workforce can be an indicator of renewal of personnel. According to the survey findings, Zimbabwe had the youngest facility-based workforce, with one quarter of health workers and half of physicians aged under 30 years (Figure 4). In contrast, in Chad none of the interviewed physicians was under 30. Although the sample size was small, with only 28 physicians included in the Chad survey, the results do suggest that the renewal of the medical workforce is not ensured for the future.

The survey also captured certain information for assessing work activities, notably on dual employment. Dual employment occurs when an employee holds two or more paid positions in more than one location. In some contexts, this may reflect a coping strategy among health personnel to overcome unsatisfactory remuneration or working conditions in order to fulfil professional and material expectations, in terms of seeking alternative ways to increase income by undertaking other forms of employment either after or during official working hours. In the assessment, health workers were asked whether they had also worked at another location (health facility or other) in the previous month. As seen in Table 4, dual employment was most frequently reported among physicians in all six countries, with as many as half – in Chad and Jamaica – reporting having a second job at the time of the interview. In Mozambique, 25% of public sector physicians reported their second job as being located in a private facility, and 12% outside of health services (results not shown). Dual employment was also found to be more common in urban areas, likely reflecting greater opportunities compared to rural areas, especially in the private sector.

**Table 4:** Percent of facility-based health workers reporting dual employment at the time of the survey, by occupation, Assessment of Human Resources for Health

	**Chad**	**Côte d'Ivoire**	**Jamaica**	**Mozambique**	**Sri Lanka**	**Zimbabwe**
Physicians	50	29	47	21	42	41
Nurses	11	7	26	17	0	7
Midwives	6	8	43	14	0	10
Others	6	5	17	19	17	8
**All health workers**	**9**	**10**	**26**	**13**	**15**	**10**

Among nurses and midwives, the rate of dual employment varied considerably across countries. In some cases this may reflect national health professional practice regulations: in Sri Lanka, for instance, it was reported that nurses did not have the right of private practice after duty hours at their government job (whereas physicians did).

## Discussion

Large cross-national differences were observed in the profile of the health workforce where the facility surveys were fielded. This may partly reflect differences in national planning for organization of the health system. It might also be a result of labour market dynamics, particularly favouring the deployment and retention of workers in urban areas or certain types of facilities. Maldistribution in the supply, deployment and composition of HRH, leading to inequities in the effective provision of health services, is an issue of social and political concern in many countries. Survey results revealed wide variations across the six countries in the distribution of workers by institutional sector, occupation, professional qualifications, age and sex.

It must be acknowledged that, although the surveys were not intended to be limited to public facilities or to any one type of facility, the results presented here should not necessarily be considered as representative of the national health workforce in any of these countries. Partly this was due to the inherent characteristics of the study design, which was limited to workers available for interview at the time of the survey, and as such excluded those who were unemployed, absent from the workplace on the day of visit (i.e. either scheduled or unscheduled absence), or working outside of health care facilities (such as at an educational institution, public health office or research laboratory). In some countries, certain types of providers are also known to provide services outside the formal health system, such as practitioners of traditional and complementary medicines.

A number of challenges also often arose during fieldwork implementation that affected the composition of the final sample. Arguably the most important challenge was a general shortage of available health personnel, especially at smaller health centres and in rural areas. In some cases the national fieldwork supervisors opted to compensate by increasing the number of larger facilities to be visited or the minimum number of workers to be interviewed per facility. How this affected the statistical representativity of the final samples remains unknown. Given this, as well as the lack of coverage of certain areas due to sociopolitical reasons in two countries (Côte d'Ivoire and Sri Lanka), we opted not to present data on the geographical distribution of providers interviewed.

Another important challenge in some countries was low response rates among providers at privately operated facilities. Involving representatives from a professional association of private providers in the survey project from the initial planning stages was cited as a crucial success factor in one country where the response rate was high. In other instances, due to work overload, some private providers indicated a preference to be surveyed by telephone rather than in person.

The study further found that a large number of health professionals, notably physicians, work in a second job, likely in order to earn additional income. Including variables on dual employment in the survey also gave some indication of work activities in the private sector, even if – as in the case of Mozambique – private facilities were not included in the final sample. Monitoring the extent and impact of dual employment has policy implications for contracting and supervision of staff, as well as equity in national regulation of health worker activities across cadres.

The level of remuneration among health service providers can be an indicator of the relative attractiveness of certain places of work compared to others. The survey included some basic questions on labour earnings; for instance, in Jamaica it was observed that physicians in the private sector tended to earn considerably more than their counterparts in public facilities (1.6 times more, results not shown). However we did not systematically present the results on occupational earnings here as, due to the study design, they did not enable comparative analysis against workers with similar characteristics outside the health sector (or even other areas within the field of health, such as research or teaching). Ideally, such analysis would be conducted by means of data from a nationally representative source, such as a population census or labour force survey [17].

## Conclusion

This study presented selected findings from the Assessment of Human Resources for Health, a survey project initiated by the World Health Organization and fielded in six low-income and middle-income countries with the aim of contributing to the evidence base to support decision-making for health workforce policies and planning. The results were presented from two perspectives: in terms of the standard survey tools developed and their application across different contexts; and in terms of the survey findings and how they can be used to inform decision-making.

While the use of standardized questionnaires offered the advantage of enhancing cross-national comparability of the eventual survey responses, some limitations were noted, especially in relation to the predefined occupational categories that did not necessarily conform to the country situations. The occupations specified in the questionnaire were largely drawn from the International Standard Classification of Occupations, a framework that enables jobs to be arranged into a hierarchical system according to the skill level and skill specialization required to carry out the tasks and duties of occupations.

Based on this framework, it was expected that most health service providers would fall into one of two major groups: "professionals" (generally well-trained workers in jobs that normally require a university or advanced-level degree for recruitment) and "technicians and associate professionals" (generally requiring skills at a tertiary non-university educational qualification level). However, it must be recognized that in some countries, the possibility of distinguishing between the two typologies of health workers remains limited. This is especially evident among nursing and midwifery personnel, whose jobs often do not fit easily into such a dichotomy.

Many titles of health workers were also recorded in the surveys that were not explicitly identified in ISCO, especially among less-specialized cadres. It may be noted that the ISCO version used for the assessment – the 1988 revision [12] – has recently been revised. A new version, adopted in 2008, overcomes some of these limitations with a greater number of cadres identified among health associate professionals (including community health workers) [18].

Likewise, large differences in self-reported educational attainment among health workers means the interpretation of the education variable needs to be addressed carefully. There are important challenges in clearly identifying the different types of training programmes for health workers from different institutions, having different entrance criteria, curricula and durations of training, and oversight regulations, then grouping them into categories that are nationally and internationally comparable.

It may be noted that the questionnaire wording itself, which was designed to capture educational attainment for becoming a practising health care provider, had certain shortcomings. It is possible that the questions did not necessarily capture the respondents' highest level of education. In addition, in contexts where a large proportion of health workers did not necessarily complete a tertiary-level or even formal health education programme, it was at times difficult to interpret and compare the results in a meaningful way without more background information on each country's education context for qualification to work in health services delivery. Future applications of the survey instrument would benefit from revising the education questions in line with internationally recommended methods for collecting and tabulating data on levels, grades and fields of education, with special attention to equivalences for persons who received their education abroad [19,20].

A particular strength of the survey instrument was the identification of each provider's sex. Many previous instruments for measuring health workforce dynamics did not include this consideration. Indeed, many (if not most) studies and strategies on the health workforce are gender-blind. However, we would argue that attention to the gender dimension is crucial to comprehensive assessment of human resources in health systems. In some contexts, access to female providers is an important determinant of women's health service utilization patterns. Omission of gender considerations may also lead to inadequate health system responsiveness to the needs of men: for example, reproductive health services are often not set up so as to encourage male involvement [21]. Future analyses of working conditions should consider factors more specifically affecting women workers, such as physical workloads, reconciling work and family, relations with clients and sexual harassment. For example, some incentives for addressing worker productivity and retention may be more favourable to female than to male workers, such as flexible working hours and leave arrangements [22].

Lastly, it is worth repeating that – although the results were useful for making valid inferences about many aspects of HRH dynamics in the countries participating in the survey programme – they should not necessarily be considered as representative of the national health workforce. Future technical cooperation initiatives for measuring and monitoring the facility-based workforce must include strengthening of national capacities to ensure that a sound and accurate sampling frame of health facilities and their staffing levels can be compiled in advance. This would entail strengthening of routine administrative human resources information systems, including the completeness and timeliness of facility staffing returns, which are often used by countries in their official reports of the health workforce situation. We recommend systematic sharing of experiences across and within countries in planning and implementation of different types of HRH data collection, both routine and periodic in nature, in order to build the global knowledge base on lessons learnt and best practices in information generation to support evidence-based decision-making.

## Competing interests

The authors declare that they have no competing interests.

## Authors' contributions

Both authors participated in the development of the survey instruments and conceptualized the study design. MRDP coordinated the survey implementation among the participating countries. NG drafted the manuscript. Both authors read and approved the final manuscript.

## Supplementary Material

Additional file 1 **Assessment of human resources for health. **Sample questionnaire for health care providers. Click here for file
