# Supplementary material for: Assessment of human resources for health using cross-national comparison of facility surveys in six countries
Source: Hum Resour Health. 2009 Mar 12;7:22. doi: 10.1186/1478-4491-7-22 (PMC2660277; doi:10.1186/1478-4491-7-22)
Supplement: Additional file 1 — Assessment of human resources for health. Sample questionnaire for health care providers. [file 1478-4491-7-22-S1.pdf]

# SAMPLE QUESTIONNAIRE FOR HEALTH CARE PROVIDERS

| IDENTIFICATION | |
|---|---|
| Name of the health facility: ____________________ | Facility code: [ ] [ ] [ ] |
| Type of facility:<br>Hospital 1<br>Health centre 2<br>Health clinic 3<br>Office 4<br>Mobile clinic 5<br>Pharmacy 6<br>Other (specify): ____________ 8 | Facility operated by:<br>Government 1<br>Private, for-profit entity 2<br>Nongovernment organization (NGO) 3<br>Charitable organization 4<br>Religious organization 5 |
| Name of district/town: ____________________ | District/town code: [ ] [ ] [ ] |
| Name of province/state: ____________________ | Province/state code: [ ] [ ] [ ] |
| Name and code of field investigator: ____________________ [ ] [ ] | Urban/rural:<br>Capital city 1<br>Other urban 2<br>Rural 3 |
| Result of final interview:<br>Completed 1<br>Partially completed 2<br>Refused 3<br>Respondent not found 4 | Date of interview:<br>Day [ ] [ ] Month [ ] [ ] Year **2002** |
| Name and code of respondent: ____________________ [ ] [ ] | Occupation of respondent:<br>Doctor 1<br>Nurse 2<br>Midwife 3<br>Auxiliary nurse 4<br>Auxiliary midwife 5<br>Pharmacist 6<br>Physiotherapist 7<br>Other health professional 8 |

***READ TO RESPONDENT:***
You have been randomly selected to be part of a survey on health and human resources, and this is why we would like to interview you. This survey is conducted by the World Health Organization and is being carried out by professional interviewers from *(name of institution)*. The survey is currently taking place in several countries around the world.

The interview will take approximately 15 minutes. I will ask you some questions about your work as a health care provider, including the practices and experiences at this and other facilities where you work. The information you provide will be used only to understand about the types of activities, payments and general working conditions of health workers in different countries.

The information you provide is totally confidential and will not be disclosed to anyone. It will be used only for research purposes. Your name, and the name and location of this facility, will be removed from the questionnaire, and only a code will be used to connect your answers with the facility without identifying you.

Your participation is voluntary and you are free to refuse to answer any question in the questionnaire. If you have any questions about this survey you may ask me or contact *(name of institution and contact details)*.

Are you willing to participate in this survey?

Agreed [ ]  Refused [ ]

| Section 1. Work status, conditions and qualifications | | | |
|---|---|---|---|
| N° | Question | **Response code** | **Skip to** |
| 101 | I would like to ask you some questions about your work as a health care provider and practices at this facility.<br><br>How would you best describe your occupation at this facility? | Medical doctor .... 1<br>Dentist .... 2<br>Pharmacist .... 3<br>Nurse .... 4<br>Midwife .... 5<br>Optometrist or optician .... 6<br>Physiotherapist .... 7<br>Medical assistant .... 8<br>Dental assistant .... 9<br>Pharmaceutical assistant .... 10<br>Nursing associate or auxiliary .... 11<br>Midwife associate or auxiliary .... 12<br>Traditional/faith healer .... 13<br>Other (specify)________ 14 | |
| 102 | What was the highest level of schooling you reached to become a practising health care provider? | Diploma .... 1<br>Associate degree .... 2<br>Baccalaureate degree .... 3<br>Master's degree .... 4<br>Doctorate .... 5<br>Other health degree (specify)______ 6<br>Other non-health (specify)______ .... 8<br>No formal degree .... 9 | <br><br><br><br><br><br>➔106<br>➔106 |
| 103 | In what year did you reach this level? | Year | |
| 104 | In what country did you reach this level? | Country of work location .... 1<br>Other country (specify)______ . 2 | <br>➔106 |
| 105 | In which school did you reach this level? | Name of school: | |
| 106 | a) How many hours a week do you usually work at this facility, excluding unpaid mealtimes and on-call hours?<br>(On-call hours are those, such as during nights and weekends, when you must be available for duty but do not have to be physically present on the hospital ward or in a clinic or laboratory except when patient needs require it.) | Hours. . . . | |
| | b) Did you work on-call hours at this facility in the last 30 days?<br><br>*IF YES:* How many on-call hours did you work here in the last 30 days? | Hours. . . .<br><br>None .... 0 | |
| 107 | What type of work do you usually do at this facility for pay?<br><br>*(CIRCLE ALL THAT APPLY)* | Direct patient care .... 1<br>Consultation with agencies/professionals .... 2<br>Administration/supervision .... 3<br>Teaching .... 4<br>Research .... 5<br>Laboratory/diagnostic procedures .... 6<br>Dispensing .... 7<br>Other (specify)______ .. 8<br>Other (specify)______ .. 9<br>Not worked for pay______ 10 | <br>➔109<br>➔109<br>➔109<br>➔109<br>➔109<br>➔109<br>➔109<br>➔109<br>➔114 |
| 108 | How many patients have you personally seen here in the last 30 days? | Number. . . .<br><br>Do not know .... 9998 | |
| 109 | How would you describe the method by which you are usually paid at this facility? | Salary .... 1<br>Fee-for-service only .... 2<br>Capitation (fixed per patient) .... 3<br>Capitation plus fees for extra services .... 4<br>Other (specify)______ .. 8 | ➔111<br>➔111<br>➔111<br><br>➔111 |
| 110 | For which types of services do you usually receive extra fees?<br><br>*(CIRCLE ALL THAT APPLY)* | Dispensed medicines .... 1<br>Other medical supplies/consumables .... 2<br>Immunizations .... 3<br>Laboratory/diagnostic procedures .... 4<br>Other (specify)______ .. 8<br>Other (specify)______ .. 9 | |

| | | | |
|---|---|---|---|
| 111 | We are interested in knowing the average income of health workers and people trained in the health field. Such information is of value when discussing health care financing options for your country. Remember that whatever you say is confidential and will be used only for research purposes.<br><br>Thinking over the past year, can you tell me what your average <u>earnings</u> from working at this facility have been? Please tell me the amount per week or per month or per year, whichever is easiest for you. | Per week ________________<br><br>Per month _______________<br><br>Per year ________________<br><br>Refuse .................................................... 9998<br>Don't know ............................................. 9999 | |
| 112 | In the past 12 months, have you experienced a <u>delay</u> in receiving your pay as scheduled from your employer? | Yes ................................................................... 1<br>No..................................................................... 2<br>Not applicable ................................................... 3 | <br>➜114<br>➜114 |
| 113 | How long would you say the delays have lasted, on average?<br><br>*(RECORD IN DAYS, WEEKS OR MONTHS AS ANSWERED)* | Number of days ___________<br><br>Number of weeks _________<br><br>Number of months _________ | |
| 114 | Do you receive any of the following additional <u>benefits</u> from working here:<br><br>*(READ EACH TYPE OF BENEFIT AND RECORD ALL ANSWERS)* | YES NO<br>Allowance for meals………………………1 2<br>Allowance for housing……………………. 1 2<br>Allowance for transportation……………..1 2<br>Paid vacations……………….……………1 2<br>Health care insurance/medical expenses.1 2 | |
| 115 | Do you regularly receive any <u>in-kind payments</u> from patients, or <u>extra payments</u> for making referrals or from other sources? | Yes ................................................................... 1<br>No..................................................................... 2 | |
| 116 | Are you currently <u>certified</u> to practise as a health care provider by any National Certifying Body? | Yes ................................................................... 1<br>No..................................................................... 2 | <br>➜118 |
| 117 | Which certifying body? | Name of body: | |
| 118 | Are you currently a member of any <u>professional association(s)</u>? | Yes ................................................................... 1<br>No..................................................................... 2 | <br>➜120 |
| 119 | Which association(s)? | Name of association(s): | |
| 120 | In the past 12 months, have you been in any <u>health/medical professional training</u> or <u>continuing education programmes</u>? | Yes ................................................................... 1<br>No..................................................................... 2 | <br>➜201 |
| 121 | For how many days (in the last 12 months) have you been on such programmes? | Number of days . . . | |
| 122 | Do you have the <u>right to strike</u>? | Yes ................................................................... 1<br>No..................................................................... 2<br>Don't know ....................................................... 3 | <br>➜201<br>➜201 |
| 123 | Have you gone on a labour strike at any time in the last 12 months, even for a short period?<br><br>*IF YES:* For <u>how many days</u> (in the last 12 months) did you go on strike? | Days. . . .<br><br>None ................................................................ 0 | |

| Section 2. Secondary employment | | | |
|---|---|---|---|
| N° | Question | **Response code** | **Skip to** |
| 201 | Now I would like to ask you some questions about your work activities at other locations.<br><br>In addition to your work at this facility, have you worked at another location in the last 30 days? | Yes ........ 1<br>No ........ 2 | <br>➔301 |
| 202 | How would you best describe this other place where you worked? | Government hospital ........ 1<br>Government health centre ........ 2<br>Government health post ........ 3<br>Government mobile clinic ........ 4<br>Other public health facility (specify) ________5<br>Private/NGO hospital ........ 6<br>Private/NGO health clinic ........ 7<br>Private/NGO mobile clinic........ 8<br>Private office ........ 9<br>Other private health facility (specify) ______10<br>Pharmacy ........ 11<br>Other non-health (specify)____________ .... 12 | |
| 203 | a) How many hours a week do you usually work at this other location, excluding unpaid mealtimes and on-call hours? | Hours. . . . | |
| | b) Did you work on-call hours at this other location in the last 30 days?<br><br>*IF YES:* How many on-call hours did you work there in the last 30 days? | Hours. . . .<br><br>None ........ 0 | |
| 204 | What type of work do you usually do at this other location for pay?<br><br>*(CIRCLE ALL THAT APPLY)* | Direct patient care........ 1<br>Consultation with agencies/professionals...... 2<br>Administration/supervision ........ 3<br>Teaching ........ 4<br>Research........ 5<br>Laboratory/Diagnostic procedures ........ 6<br>Dispensing ........ 7<br>Other (specify)______________________ .. 8<br>Other (specify)______________________ .. 9<br>Not worked for pay____________________10 | <br><br><br><br><br><br><br><br><br>➔208 |
| 205 | How would you describe the method by which you are usually paid at this other location? | Salary ........ 1<br>Fee-for-service only ........ 2<br>Capitation (fixed per patient) ........ 3<br>Capitation plus fees for extra services ........ 4<br>Other (specify)______________________ .. 8 | ➔207<br>➔207<br>➔207<br><br>➔207 |
| 206 | For which types of services do you usually receive extra fees there?<br><br>*(CIRCLE ALL THAT APPLY)* | Dispensed medicines ........ 1<br>Other medical supplies/consumables ........ 2<br>Immunisations........ 3<br>Laboratory/Diagnostic procedures ........ 4<br>Other (specify)______________________ .. 8<br>Other (specify)______________________ .. 9 | |
| 207 | What are your average earnings from working at this second location? Please tell me the amount per week or per month or per year, whichever is easiest for you. (Remember that whatever you say is confidential and will be used only for research purposes.) | Per week ________________<br><br>Per month _______________<br><br>Per year ________________<br><br>Refuse ........ 9998<br>Don't know ........ 9999 | |
| 208 | Do you receive any of the following additional benefits from working there?<br><br>*(READ EACH TYPE OF BENEFIT AND RECORD ALL ANSWERS)* | YES NO<br>Allowance for meals……………………1 2<br>Allowance for housing…………………… 1 2<br>Allowance for transportation……………..1 2<br>Paid vacations…………………..…………1 2<br>Health care insurance/medical expenses.1 2 | |

| Section 3. Occupational mobility | | | |
|---|---|---|---|
| N° | Question | **Response code** | **Skip to** |
| 301 | I would like to ask a few questions about your work experience.<br><br>How many years of experience do you have in practice as a health care provider? | Years. . . . | |
| 302 | For how long have you been working at this facility here?<br><br>*(RECORD IN WEEKS, MONTHS OR YEARS AS ANSWERED)* | Number of weeks __________<br><br>Number of months _________<br><br>Number of years ___________ | *If less than one month, or same as total years experience* ➜401 |
| 303 | How would you describe the last place where you worked before coming to this facility? | Government hospital ...................................... 1<br>Government health centre............................. 2<br>Government health post ................................. 3<br>Government mobile clinic ............................... 4<br>Other public health (specify) _____________ 5<br>Private/NGO hospital...................................... 6<br>Private/NGO health clinic ............................... 7<br>Private/NGO mobile clinic .............................. 8<br>Private office ................................................... 9<br>Other private health (specify) ___________10<br>Pharmacy...................................................... 11<br>Other non-health (specify)______________ 12<br>Same as current secondary place................ 13 | ➜401 |
| 304 | What type of work did you usually do at that last location for pay?<br><br>*(CIRCLE ALL THAT APPLY)* | Direct patient care........................................... 1<br>Consultation with agencies/professionals...... 2<br>Administration/supervision ............................. 3<br>Teaching ......................................................... 4<br>Research.......................................................... 5<br>Laboratory/diagnostic procedures.................. 6<br>Dispensing ...................................................... 7<br>Other (specify)_______________________ .. 8<br>Other (specify)_______________________ .. 9<br>Not applicable/Was not paid ......................... 10 | |
| 305 | Where was your former work located? | In the same city/rural district........................... 1<br>In a different city ............................................ 2<br>In a different rural district................................ 3<br>In another country (specify)______________ 4 | |

| Section 4. Sociodemographic characteristics | | | |
|---|---|---|---|
| N° | Question | **Response code** | **Skip to** |
| 401 | Lastly, some additional information for use in the statistical interpretation of your responses:<br><br>*RECORD SEX AS OBSERVED* | Male ................................................................ 1<br>Female ........................................................... 2 | |
| 402 | What is your date of birth? | Month<br><br>Year | |

**Contacts at World Health Organization Headquarters, Geneva, Switzerland**
**Dr Alexandre Goubarev**: Fax: +41 22 791 4747; Email: goubareva@who.int
**Dr Mario Dal Poz:** Fax: +41 22 791 4747; Email: dalpozm@who.int
